# Editorial: Recent Advances in the Study of the Host-Fungus Interaction

**DOI:** 10.3389/fmicb.2016.01694

**Published:** 2016-10-26

**Authors:** Héctor M. Mora-Montes, Attila Gacser

**Affiliations:** ^1^Departamento de Biología, División de Ciencias Naturales y Exactas, Universidad de GuanajuatoGuanajuato, Mexico; ^2^Department of Microbiology, University of SzegedSzeged, Hungary

**Keywords:** *Candida*, dermatophytes, *Paraccocidioides*, *Aspergillus*, *Cryptococcus*, cell wall, melanin, *Histoplasma*

Fungal infections are worldwide distributed and are associated with high morbidity rates, and in immunocompromised populations, are usually life-threatening conditions. The host-fungus interaction is critical for tissue adhesion, colonization and damage. Here, we provide a series of comprehensive review and original papers dealing with both aspects of this interaction: the role of virulence factors and cell wall components during contact with host cells, and the recent advances in the study of the antifungal immune response.

The fungal cell wall, along with the capsule and secreted components, is a relevant organelle during interaction with the host, and is the source of most of the pathogen-associated molecular patterns (PAMPs) recognized by immune cells. Melanin is one of its components found in many human pathogens, and plays a significant role in protecting the fungal cell against extracellular insults, including damage by immune cells. Here, Nosanchuk et al., provide an updated view of melanin synthesis and interaction with other wall components, with emphasis in the information derived from new analytical approaches.

Dermatophytes are the most frequent fungal pathogens causing human infections. Although most of them are related to superficial and not harmful infections, they can cause chronic dermatophytosis in immunocompromised population. Here, de Sousa et al., explore the mechanism underlying this infection, but in immunocompetent patients. Their results indicate that macrophages and neutrophils from patients with the chronic disease showed bias to generate a strong anti-inflammatory response. Since this observation was not found in patients with superficial skin infections, these data stress a differential host-dermatophyte interaction in both superficial and deep tissues.

*Paracoccidioides* spp. is the etiological agent of paracoccidioidiomycosis, a relevant systemic fungal infection in South America. The disease is mostly associated to rural workers, malnutrition, poor hygiene and chronic alcohol consumption and smoking. Here, de Oliveira et al. summarize the most important finding regarding cell surface adhesins, highlighting their role in virulence. Furthermore, authors explore the importance of the morphological switching during host colonization and tissue damage, as the filament to yeast transition is accompanied with changes in the cell wall and surface molecules that have a direct impact on the host-fungus interaction. As a new therapeutic and prophylactic approach to control paracoccidioidiomycosis, Bueno et al., describe the use of polyclonal antibodies against acidic glycosphingolipids to opsonize *P. brasiliensis* yeast cells. This strategy successfully increased fungal phagocytosis and had a positive impact in the murine model of intratracheal infection, with lower fungal burdens and tissue damage.

*Histoplasma capsulatum* is the causative agent of a worldwide distributed systemic disease that often affects lungs. As in the case of other fungal pathogens, phagocytic cells play a key role in controlling *H. capsulatum* yeast cells, being readily phagocytosed upon detection. Pitangui et al. demonstrate that, upon phagocytosis, *H. capsutalum* yeast cells aggregate around the nucleus of murine alveolar macrophages, forming a crown-like structure that stimulates macrophage apoptosis, thus describing a new mechanism to evade control by these immune cells.

Systemic cryptococcosis, candidiasis, and aspergillosis account for most of the opportunistic infections caused by fungi. Infections associated to *Cryptococcus neoformans* often affect immunocompromised patients; while the closely related species *Cryptococcus gattii* mainly affects healthy individuals and displays a decreased tropism to the central nervous system. Leopold Wager et al. describe the most relevant finding about the immune response stimulated by *Cryptococcus* spp., with especial emphasis in phagocytic cells. They discuss the survival and escaping mechanisms evolved for these pathogens to survive the macrophage strike, pointing out to the capsule presence as a mechanism to prevent phagocytosis, and phospholipase B as one of the mechanisms to survive within phagosomes. Finally, they also discuss the main PAMPS and receptors that allow recognition of *Cryptococcus* by immune cells and the potential of immunomodulatory therapies for cryptococcosis control. Reinforcing the central role of macrophages in *Cryptococcus* control, DeLeon-Rodriguez and Casadevall discuss the strategies involved in the survival of *C. neoformans* within the phagolysosome, with special emphasis in the observation that the outcome of the yeast-macrophage interaction depends on the extent of the damage to the phagosomal membrane.

Invasive aspergillosis is also considered as a common infection in immunocompromised patients, and it is usually caused by *Aspergillus fumigatus*. Here, Espinosa and Rivera present a thoroughly review about the receptors involved in the immune recognition of this pathogen, along with the relevance of most of the innate immune cells during the *Aspergillus*-host interaction.

Systemic candidiasis is one of the most frequent nosocomial fungal infections and is often associated to immunosuppression, tissue colonization and damage by *Candida albicans.* This organism forms part of the human microbiota, and has to adapt to several environmental conditions prevailing in human mucosa. Here, Prieto and Pla, using fluorescent *C. albicans* cells demonstrate that this organism rapidly adapts to the mouse gut, increasing the cell fitness once they proliferated for long colonizing periods; a fact that should be taken in consideration during candidiasis treatment. In addition of this, *C. albicans* needs to adhere to host tissues and cells in order to colonize and invade. Hoyer and Cota put together most of the relevant information generated in the last 20 years about one of the most studied adhesins family in *C. albicans*, the ALS family; with emphasis in the structural determinants that control ligand binding, aggregation and invasion of host cells.

Members of the *Candida parapsilosis* complex are other fungal species usually associated to candidiasis. Estrada-Mata et al., demonstrate that these organisms have subtle differences in the cell wall organization when compared to *C. albicans*, which are likely related with a differential recognition when incubated with human peripheral blood mononuclear cells. At difference of *C. albicans, C. parapsilosis sensu lato* stimulates high cytokine levels in a *O*-mannan dependent mechanism. Moreover, Tóth et al. show that two *C. parapsilosis* strains stimulate a similar phagocytic response, but differences in the human macrophage migration and cell engulfment were observed; underlining the fact of heterogeneity in the host-fungus interaction given only by different isolates of one fungal species. Finally, authors demonstrate that secreted lipases play a significant role precluding macrophage uptake.

## Author contributions

Both authors wrote and approved the manuscript.

## Funding

This work was funded by NKFIH NN 113153, GINOP-2.3.2.-15-2016-00015, GINOP-2.3.3-15-2016-00006, CONACYT (ref. CB2011/166860; PDCPN2014-247109), and Red Temática Glicociencia en Salud (CONACYT-México).

### Conflict of interest statement

The authors declare that the research was conducted in the absence of any commercial or financial relationships that could be construed as a potential conflict of interest.

